# Evaluation of a New ‘Mix-In’ Style Glycomacropeptide-Based Protein Substitute for Food and Drinks in Patients with Phenylketonuria and Tyrosinemia

**DOI:** 10.3390/nu15163598

**Published:** 2023-08-17

**Authors:** Marta Delsoglio, Rebecca Capener, Anita MacDonald, Anne Daly, Catherine Ashmore, Sarah Donald, Lisa Gaff, Louise VanDorp, Rachel Skeath, Charlotte Ellerton, Camille Newby, Georgina Dunning, Clare Dale, Inderdip Hunjan, Lucy White, Heather Allen, Gary P. Hubbard, Rebecca J. Stratton

**Affiliations:** 1 Research and Innovation, Nutricia Ltd., White Horse Business Park, Trowbridge BA14 0XQ, UK; 2 Dietetic Department, Birmingham Children’s Hospital, Birmingham B4 6NH, UK; 3 Cambridge University Hospitals NHS Foundation Trust, Cambridge CB2 0QQ, UK; 4 Great Ormond Street Hospital for Children NHS Foundation Trust, London WC1N 3JH, UK; 5 University College London Hospitals NHS Foundation Trust, London WC1N 3BG, UK; 6 Bristol University Hospitals NHS Foundation Trust, Bristol BS1 3NU, UK; 7 Queen Elizabeth Hospital, Birmingham B15 2TH, UK; 8 Bradford Teaching Hospitals, NHS Foundation Trust, Bradford BD5 0NA, UK; 9 Sheffield Children’s NHS Foundation Trust, Sheffield S10 2TH, UK; 10 Faculty of Medicine, University of Southampton, Southampton SO16 6YD, UK

**Keywords:** glycomacropeptide, phenylketonuria, tyrosinemia, PKU, TYR, protein substitute

## Abstract

(1) Background: Poor palatability, large volume, and lack of variety of some liquid and powdered protein substitutes (PSs) for patients with phenylketonuria (PKU) and tyrosinemia (TYR) can result in poor adherence. This study aimed to evaluate a new unflavoured, powdered GMP-based PS designed to be mixed into drinks, foods, or with other PSs, in patients with PKU and TYR. (2) Methods: Paediatric and adult community-based patients were recruited from eight metabolic centres and prescribed ≥1 sachet/day (10 g protein equivalent (PE)) of the Mix-In-style PS over 28 days. Adherence, palatability, GI tolerance, and metabolic control were recorded at baseline and follow-up. Patients who completed at least 7 days of intervention were included in the final analysis. (3) Results: Eighteen patients (3–45 years, nine males) with PKU (*n* = 12) and TYR (*n* = 6) used the Mix-In-style PS for ≥7 days (mean 26.4 days (SD 4.6), range 11–28 days) alongside their previous PS, with a mean intake of 16.7 g (SD 7.7) PE/day. Adherence was 86% (SD 25), and GI tolerance was stable, with *n* = 14 experiencing no/no new symptoms and *n* = 3 showing improved symptoms compared to baseline. Overall palatability was rated satisfactory by 78% of patients, who successfully used the Mix-In-style PS in various foods and drinks, including smoothies, squash, and milk alternatives, as a top-up to meet their protein needs. There was no concern regarding safety/metabolic control during the intervention. (4) Conclusions: The ‘Mix-In’-style PS was well adhered to, accepted, and tolerated. Collectively, these data show that providing a flexible, convenient, and novel format of PS can help with adherence and meet patients’ protein needs.

## 1. Introduction

Phenylketonuria (PKU) and tyrosinemia (TYR) are rare inherited disorders of the amino acid metabolism, which need lifelong management to avoid severe cognitive impairment and disability [1,2].

The dietary management of PKU and TYR is complex and burdensome for patients and their families. It involves the restriction of dietary protein intake, supplementation with Phe- or Phe- and Tyr-free protein substitutes, respectively, and can include the consumption of low-protein food products, to ensure optimal metabolic control, whilst meeting the nutrient requirements for growth and normal functioning [2,3,4,5,6,7,8,9,10]. As Phe and Tyr are present in significant quantities in nearly all dietary proteins, this usually requires a very-low-protein diet. The low-Phe and -Tyr diets prescribed for patients with classical PKU and TYR are limited to a narrow selection of food groups, including fruits, most vegetables, sugars, pure fats, and special low-protein foods. Most high-protein foods, such as dairy products, meats, legumes, nuts, and most grains, are typically avoided due to the high content of Phe and Tyr, which creates challenges both nutritionally and in terms of food palatability and variety [11,12]. A protein substitute is essential for most patients with PKU and TYR, supplying classical patients with 75% to 85% of daily protein needs [11,13] and as an essential source of nutrients for which patients are at risk of being deficient due to their restrictive diet: vitamins (e.g., B12, D), minerals (e.g., calcium, selenium), and long-chain polyunsaturated fats (e.g., DHA) [11,14]. Consequently, poor adherence to protein substitutes can lead not only to high blood Phe levels but also to malnutrition [14,15]. Protein substitutes also improve Phe tolerance and optimize metabolic control by suppressing blood Phe levels [15]. This is particularly important during illness and trauma, where protein substitutes have a protective role by counter-acting protein catabolism.

Adherence to diet and the restriction of Phe and Tyr intake in those with PKU and TYR are essential, not only in the development windows of infancy and childhood but should be continued for life in order to optimise health outcomes and prevent lifelong mental impairments [15,16]. During infancy and early childhood, dietary adherence is usually adequate for optimal metabolic control. However, dietary adherence becomes more difficult when growing up due to the restrictive diet and higher volumes of protein substitutes required, with patients commonly going “off diet” during adolescence and adulthood, often associated with disengagement from clinical review [17,18,19,20]. The deterioration of treatment adherence with chronic diseases is a well-recognised phenomenon [17] and is influenced by physical, social, and emotional changes that occur during adolescence [21]. Adolescents and adults commonly have difficulty in adequately adhering to strict treatment regimens that they feel constitute an unnecessary burden and set them apart from their peers [22]. Walter et al. (2002) found that adherence to Phe restriction worsens with age, and by 15–18 years of age, up to 79% of British PKU patients have blood Phe levels outside the recommended limits [15]. Similarly, in a more recent survey (2018), Ford et al. found the number of participants following a low-Phe diet decreased with age, and only 57% of adults said they were on a prescribed low-Phe diet [23]. In the US, patients’ adherence to dietary recommendations was also found to be age-dependent, decreasing from 88% in the 0–4-years age group to 33% in adults ≥30 years [24]. Similarly, in the TYR population, González-Lamuño et al. observed that dietary adherence was unsatisfactory in more than half of all patients with TYR, and 69% of non-dietary adherents were between 12 and 18 years of age [18]. Poor palatability and the undesirable texture and smell of protein substitutes are a challenge for patients with PKU and TYR, which must be taken regularly (ideally ≥ 3 times daily) as part of their dietary management [16]. Patients also experience anxiety about taking their protein substitute and guilt related to poor adherence to dietary restrictions or supplement intake [25].

Although the foundations of dietary control in PKU and TYR have not really changed since their introduction in 1952, protein substitutes have evolved [26]. Historically, the protein source of protein substitutes has been free amino acids. More recently, casein glycomacropeptide (GMP), a large peptide (64 amino acids) produced during cheese making, has emerged as a uniquely suited protein source for PKU and TYR as it contains minimal Phe (2.5–5 mg Phe/g protein) [27]. GMP has been used to improve the taste and variety of protein substitutes for patients with PKU and TYR, though some free amino acids still have to be added to GMP-based protein substitutes (GMP-PSs) as GMP does not contain all essential amino acids. Sensory analysis studies in individuals with PKU have shown that GMP-PSs are acceptable alternatives to Phe-free amino acids and may improve adherence and help patients with PKU and TYR and their families to manage dietary challenges [27,28,29,30,31] while maintaining metabolic control [32,33]. Further innovation in dietary approaches to improve adherence to diet, such as enhancing the palatability and variety of the low-Phe and low-Phe and -Tyr diets for individuals with PKU and TYR, are still needed. The majority of commercially available protein substitutes are ready-to-drink liquid or powder-based drinks reconstituted with water. Although Phe-free amino acid tablets have been shown to be efficacious [34], in practice, only a relatively small number of patients are able to integrate the tablets into their dietary regimen. Recently, powdered ready-to-mix, unflavoured, and odourless GMP-PSs have been developed, which offer a novel format (Mix-In style) that can be combined with drinks and foods. The aim of this study was to evaluate the adherence, gastrointestinal (GI) tolerance, and palatability of this new type of protein substitute in paediatric and adult community patients with PKU and TYR, as well as any changes in metabolic control.

## 2. Materials and Methods

### 2.1. Study Design and Ethics

This was a prospective, interventional, multi-centre case study series. From baseline, each patient was prescribed at least one sachet of the Mix-In-style GMP-based protein substitute for 28 days. Outcome measurements were taken at baseline and at the end of the intervention period. The study protocol was approved by the South West-Central Bristol Research Ethics Committee and was registered at clinicaltrials.gov (NCT05062226). UK Health Research Authority (HRA) approval and local NHS R&D/site approval were obtained from all sites involved. The study was conducted in accordance with the Declaration of Helsinki and Good Clinical Practice (GCP) guidelines. All patients or patients’ parents provided written informed consent before any study-related procedures were performed.

### 2.2. Recruitment and Study Population

Paediatric and adult patients from 8 metabolic centres in the UK were screened using the following inclusion criteria: male or female, over 3 years of age, diagnosed with classical or variant type PKU, or TYR (as appropriate), adherent in taking at least one protein substitute dose providing at least 10 g protein equivalents for at least 1 month prior to trial commencement, prescribed daily Phe or Phe and Tyr allowance for PKU or TYR, respectively, informed consent provided from patient and/or from parent/caregiver, as appropriate. Patients were excluded from the study if they were: pregnant or lactating, requiring parenteral nutrition, presence of major hepatic or renal dysfunction, participation in other studies within 1 month prior to entry of this study, allergy to any of the study product ingredients, and investigator concerns around willingness/ability of patient or parent/caregiver to adhere with protocol requirements.

### 2.3. Study Intervention and Study Product

At baseline, medical and nutritional history, anthropometrics, gastrointestinal tolerance, adherence, and palatability of current protein substitutes were recorded by the managing dietitian. Each patient was then prescribed at least one sachet of Mix-In-style unflavoured GMP-PS (PKU or TYR GMPro Mix-In, Nutricia Ltd., Trowbridge, UK) for 28 days, with the prescription being specified on an individual basis by the dietitian responsible for the patient’s nutritional management. Each serving of study product (12.5 g sachet) provided 10 g protein equivalent (PE) and 42 kcal and contained essential and non-essential amino acids, a small amount of carbohydrates (0.43 g), and some minerals (see Table A1 for the full nutritional profile). The study product could be mixed into drinks, low-protein foods, or another protein substitute, alongside or as a replacement for patients’ existing prescribed protein substitutes. Full preparation, storage, safety instructions, and examples of suitable recipes were provided to patients before commencing the intervention to ensure its correct use. 

### 2.4. Outcomes

#### 2.4.1. Adherence to the Intervention

Adherence to the recommended intake of protein substitute was assessed by the managing dietitian for the baseline protein substitute, as well as for the study product. The dietitian asked the patient or parent (if applicable) how much of the study product (all, 75%, 50%, 25%, none) was taken on an average day over the study period. If the full prescription was not taken, patients/parents were asked to describe the reasons why.

#### 2.4.2. Gastrointestinal Tolerance

The intensity (none, mild, moderate, or severe) of gastrointestinal (GI) symptoms (diarrhoea, constipation, nausea, vomiting, abdominal discomfort, bloating, flatulence, and burping) in the previous 24 h was recorded by the managing dietitian at baseline and end of the study, using a standardised questionnaire. The dietitian also recorded if these symptoms reflected the typical GI tolerance on an average day during the study period.

#### 2.4.3. Palatability and Convenience

At the end of the study, the managing dietitian asked the patient or parent (if applicable) to rate the study product using a 5-point Likert scale (great, good, ok, bad, terrible) regarding overall acceptability and the following product characteristics: appearance, smell, taste, texture (mouthfeel), aftertaste, smell of breath after taking, ease of mixing, and ease of taking.

#### 2.4.4. Metabolic Control and Safety

To evaluate metabolic control at baseline and end of intervention, the managing dietitian recorded blood Phe and Tyr concentration obtained from dried blood spots as part of routine clinical management, if available. Furthermore, any adverse and serious adverse events were recorded throughout the study period.

### 2.5. Statistics

Descriptive statistics are presented on the characteristics of the study population and the findings for adherence, GI tolerance, and acceptability. Changes in blood Phe control between baseline and study end, when available, were compared for each subject and with age-specific target treatment ranges. Patients who completed a minimum of 7 days of intervention were included in the final analysis.

## 3. Results

### 3.1. Recruitment and Patient Characteristics

A total of 26 patients (19 PKU and 7 TYR) were considered eligible and consented to take part in the study. Two patients with PKU tried and refused the study product after one day, and five dropped out after 2–3 days because they found the study product less practical than their existing liquid protein substitute (*n* = 2) or did not enjoy the neutral taste (*n* = 3). One patient with TYR refused the study product, possibly due to his feeding difficulties and dysphagia. Thus, a total of 18 patients (12 PKU and 6 TYR) took the study product for at least 7 days (mean 26.4 (SD 4.6), range 11–28 days) and were included in the analysis. Baseline characteristics are shown in Table 1. Patients’ ages ranged from 3 to 45 years (mean 18, SD 14), and nine patients (50%) were male. Patients had a mean baseline weight of 55.9 kg (SD 22.6) and mean protein requirements of 65.6 g/day (SD 19.4). At baseline, nine patients were prescribed a liquid protein substitute, eight patients a powdered protein substitute, and *n* = 1 both liquid and powder formats. Fifteen patients had Phe and Tyr blood spots recorded. At baseline, Phe levels were within the target range in most patients with PKU (7/9) and above target in *n* = 2. In TYR patients, Phe and Tyr levels fluctuated around the target in four patients and were above target in *n* = 2. 

Patients were prescribed 1–4 sachets per day of the Mix-In study product, providing a mean of 16.67 g (SD 7.67) PE per day, as a top up alongside their current protein substitutes to meet their protein needs.

### 3.2. Adherence to the Protein Substitute

Mean daily adherence to the baseline protein substitute was 89% (SD 23), with most patients (*n* = 15/18) consuming 75–100% of the amount prescribed and three patients struggling to achieve the full intake (25–50% compliance) due to perceived poor palatability. Mean daily adherence to the study product (86% (SD 25)) did not differ significantly to adherence to the baseline protein substitute (*p* = 0.62), with most patients (*n* = 14/18) consuming 75–100% of the amount prescribed and four patients struggling to achieve the full intake (25–50% compliance) due to preference for or the perceived convenience of their baseline protein substitute Mean adherence to the study product was similar in patients with PKU (85% (SD 27) and patients with TYR 88% (SD 21).

### 3.3. Gastrointestinal Tolerance

**Figure 1:**
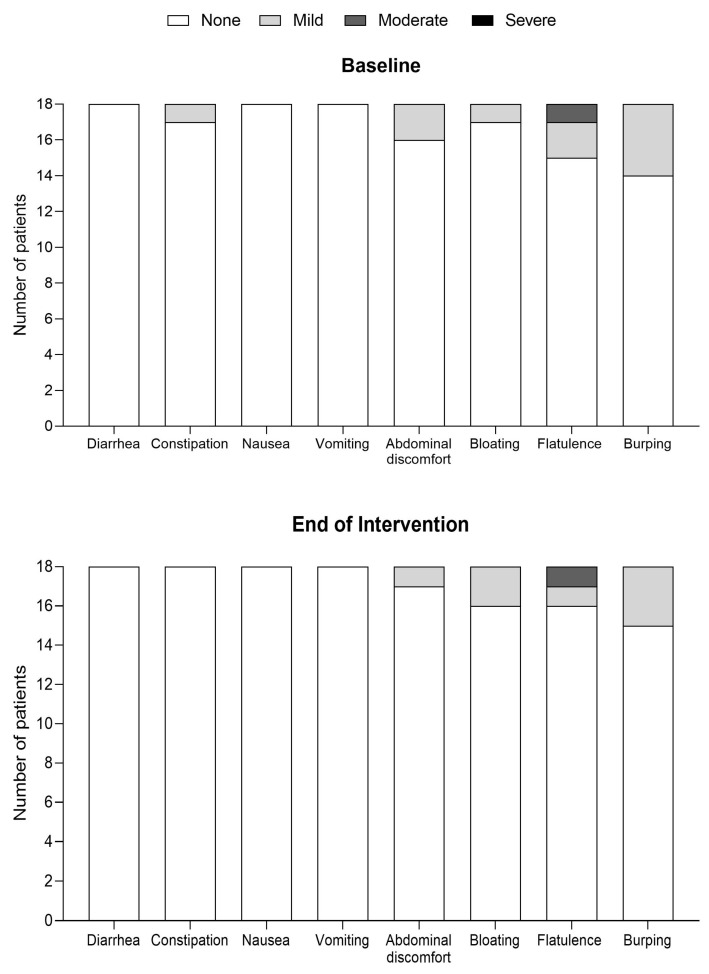
Gastrointestinal symptoms recorded at baseline and end of intervention with the Mix-In protein substitute (*n* = 18).

Most GI symptoms were absent at baseline, with a few occurrences of mild–moderate symptoms reported for constipation (1/18), abdominal discomfort (2/18), bloating (1/18), flatulence (3/18), and burping (4/18). At the end of intervention, whilst taking the study product, GI tolerance remained stable, with 14 patients experiencing no/no new symptoms, 3 patients reporting the resolution of constipation, abdominal discomfort, flatulence, and burping compared to baseline, and 1 patient experienced some unrelated flatulence (Figure 1). GI symptoms recorded at the end of intervention were reported to reflect typical GI tolerance on an average day during the study period.

### 3.4. Palatability and Convenience

Overall acceptability of the study product was rated as satisfactory (‘Ok’, ‘Good’, or ‘Great’) by 78% of patients, based on all attributes, which included appearance (83%), smell (89%), taste (72%), texture/mouthfeel (83%), aftertaste (83%), smell of breath after taking (94%), ease of mixing (72%), and ease of taking (78%). When considered separately, 67% of PKU patients rated overall palatability as satisfactory, compared with 100% of patients with TYR. Patients successfully used the study product in various foods and drinks, including smoothies, juice and squash (*n* = 7), strong-tasting foods (*n* = 2), drinks (*n* = 1), lemonade and ice cream (*n* = 1), porridge (*n* = 1), milkshake (*n* = 1), crushed fruit salad (*n* = 1), custard (*n* = 1), plant-based milk alternative (*n* = 1), and low-protein milk (*n* = 1). Two parents reported that they were satisfied with the flavourless nature of the study product and being able to mix it into food and drinks without their children knowing.

### 3.5. Metabolic Control and Safety

**Figure 2:**
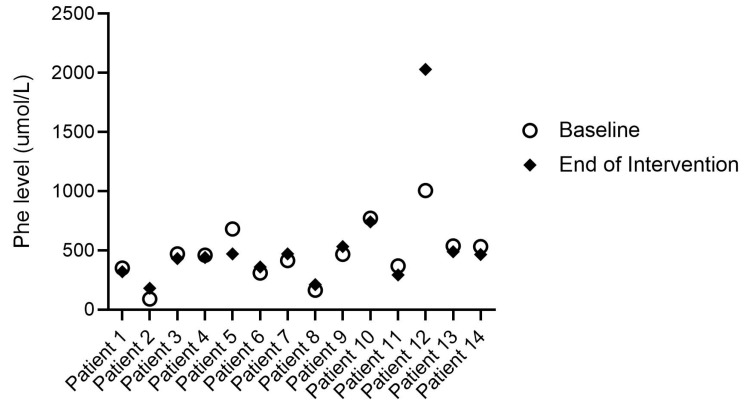
Individual Phe levels in patients with PKU at baseline and end of intervention (*n* = 9).

In patients with PKU, baseline Phe blood spot results were within the target range for their age group in *n* = 7 and above range in *n* = 2 (mean: 383 µmol/L (SD 171)). At the end of intervention, the mean Phe level (335 µmol/L (SD 184)) was maintained. Individual Phe levels at baseline and the end of intervention are presented in Figure 2; metabolic control was maintained in most PKU patients (7/9), it improved in *n* = 1, and worsened, while still being within the target range (<600 µmol/L), in 1 young patient due to poor adherence to diet during a holiday from school. 

**Figure 3:**
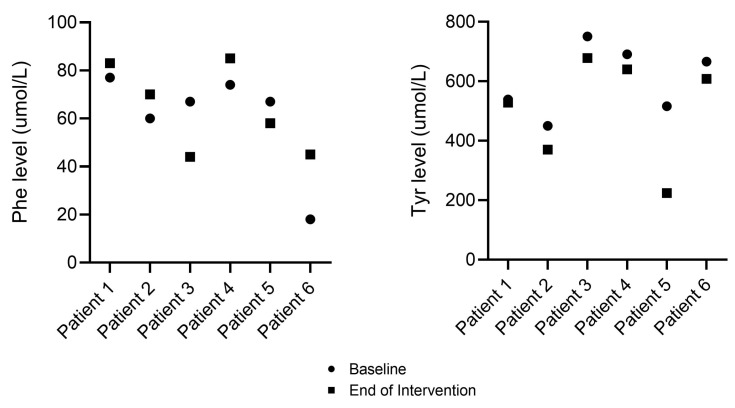
Individual Phe and Tyr levels in patients with TYR at baseline and end of intervention (*n* = 6). No adverse or serious adverse events were reported during the study period.

In patients with TYR, baseline Phe and Tyr blood spot results fluctuated around the target range for their age group in *n* = 4 and above the target range in *n* = 2 (mean Phe: 61 µmol/L (SD 22), mean Tyr: 602 µmol/L (SD 117)). At the end of intervention, mean Phe and Tyr levels (64 µmol/L (SD 18) and 508 µmol/L (SD 177)) were maintained. Individual Phe and Tyr levels at baseline and end of intervention are presented in Figure 3; metabolic control was maintained in five patients out of six and improved in *n* = 1 (patient n5). 

## 4. Discussion

Adherence to diet therapy in PKU and TYR is a well-known but complex challenge, particularly in adolescence and adulthood. Many factors are at play, including the poor palatability, large volume, and lack of variety of protein substitutes [23]. Interventions to improve dietary adherence in patients with PKU and TYR have been limited. Thus, this study aimed to evaluate a new powdered GMP-PS for PKU and TYR that can be consumed in a novel way by mixing into drinks, low-protein food, or protein substitutes, in adult and paediatric patients. 

Overall, the results showed that the study product was well adhered to, tolerated, and patients reported good palatability. Patients successfully used the Mix-In protein substitute in various foods and drinks while maintaining metabolic control compared to baseline.

Measuring adherence to treatment in PKU and TYR is not straightforward, and there is not currently a single measure able to determine all facets of adherence with treatment, there is also no consensus about the definition of acceptable adherence with any specific measures [16]. Different measures to assess adherence are used in the clinical setting, including laboratory methods (percentage of blood Phe concentrations within target range, annual mean or median blood Phe concentration); quantitative nonlaboratory methods (percentage of blood samples returned by patients and their carers; attendance at outpatient clinics, self-reported dietary records, protein substitute prescription records); and qualitative surveys or patient self-ratings through interviews with healthcare professionals [16]. 

Our study assessed adherence to the novel GMP-based powdered protein substitute by recording the amount consumed vs. the amount prescribed by the managing dietitian over the intervention period, which has been reported as a valid tool to determine compliance [35,36]. Patients with PKU and TYR showed high adherence (85%) to the study product, with most patients (78%) consuming the amount prescribed and with no significant difference compared to adherence to their baseline protein substitute (88%).

Previous studies have reported poor adherence to diet in patients with PKU and TYR and underlined the challenges related to lifelong adherence to a natural protein-restricted diet and the consumption of protein substitutes [24,37,38,39,40,41,42].

Cazzorla et al. [38] showed low adherence with dietary recommendations among adult patients with PKU, with less than half (42%) claiming full adherence, increased consumption of natural protein sources, and reduced daily use of amino acid supplements (<4–5 times/day in 82% patients). Similarly, Cotugno et al. [39] found an adherence to dietary prescriptions slightly in excess of 50% (56.1%), close to that commonly reported for adherence to drugs. More recently, a survey in six specialist metabolic centres reported the level of adherence in adult patients on prescribed treatment was considered low/moderate, not exceeding 65% [43].

Strategies to improve the taste and acceptability of protein substitutes are important, as a lack of palatability and ease of use for protein substitutes have been associated with poor adherence [44]. The positive adherence found in our study could be explained by several intrinsic factors associated with the study product. Firstly, it was designed to be combined with regular foods, and this may have been more acceptable to patients rather than adding a separate protein supplement drink to their diet. Secondly, the study product was a flavourless powder designed to minimise the impact on the organoleptic characteristics of the food or drink it was combined with. This was reported to be useful for parents who were able to add the study product to foods without their child knowing. This may explain the positive acceptability ratings observed for most patients (78%). Our palatability finding is also consistent with previous studies that reported GMP-based protein substitutes were well accepted, better tasting, and added variety to the low-Phe diet compared to patients’ usual AA-based protein substitutes [28,45].

In this study, patients with TYR seemed to accept the study product better than those with PKU (100% vs. 67%). This may have been driven by the more limited variety of protein substitutes available for patients with TYR. The younger age of the TYR population in this study, all of whom were children, may have also influenced the positive results compared to patients with PKU.

The Mix-In protein substitute was also well tolerated in this study, with GI symptoms remaining stable in 78% of patients, who experienced no/no new symptoms over the intervention period, and improving in 17% of patients, who showed resolution of GI symptoms compared to baseline. Tolerance is another factor that can impact adherence; indeed, some protein substitutes have been shown to cause GI upset, abdominal pain, diarrhoea, and constipation and, therefore, represent a challenge for patients to take the full dose prescribed [8].

In our study, blood Phe and Phe and Tyr levels and metabolic control were assessed in 15 patients who had a blood spot while taking the study products. Most PKU (89%) and all TYR patients showed maintained or improved metabolic control at the end of intervention compared to baseline. Previous studies in PKU have reported a high percentage of blood Phe concentrations that are above the target ranges, particularly in teenagers and adults, due to poor dietary adherence [15,46]. In children, a few studies have reported that GMP-based products may cause a deterioration in metabolic control and an increase in blood Phe concentration [47]. The stable metabolic control during the intervention period in this study suggests that the addition of the study product as an adjunct to existing protein substitutes in the diets of younger patients’ with PKU and TYR did not negatively affect Phe and Phe and Tyr levels; however, cautious monitoring is required if higher doses are prescribed.

### Limitations

Our study had several limitations. Firstly, the sample size was quite small and heterogeneous, as it included both adult and paediatric patients. Due to the rarity of the condition, the pool of patients is limited, and many were already taking part in other trials. Secondly, some patients who consented to take part in the study (37% PKU and 14% TYR) were excluded from final analysis as they dropped out after a few days. Early dropouts are common in this group of patients, mainly due to resistance to product switching, established taste preferences, and the specific characteristics of each protein substitute. Indeed, preferences for PS are highly individual, and patients may be resistant to change, especially when they are established on a preferred product or combination of products. Finally, another limitation could be that patients were not requested to send in additional blood samples specifically for the purpose of the study, i.e., at baseline and end of intervention; however, our pragmatic approach to collect the most recent results before entering the study and during the study period as part of usual care/monitoring eased the burden on patients and reflected real clinical practice.

## 5. Conclusions

The results of this study demonstrated that a Mix-In-style novel-format GMP-based flavourless powdered protein substitute was well adhered to, tolerated, and accepted, with paediatric and adult patients mixing it in various foods and drinks. The study product helped maintain stable metabolic control in combination with existing protein substitutes and may be a useful adjunct in the dietary management of patients with PKU and TYR.

## Figures and Tables

**Table 1:** Patient population baseline characteristics.

	PKU (*n* = 12)	TYR (*n* = 6)	Total (*n* = 18)
Age *, years, mean (SD) [range]	22 (15) [3–45]	9 (3) [3–12]	18 (14) [3–45] *
Height, cm, mean (SD)	162.2 (24.6)	134.9 (29.2)	155.4 (27.7)
Weight, kg, mean (SD)	64.0 (20.5)	36.6 (14.7)	55.9 (22.6)
Protein requirements, g/day, mean (SD)	71.0 (18.8)	54.7 (17.1)	65.6 (19.4)
Protein from PS, g/day, mean (SD)	65.4 (16.7)	48.3 (11.2)	59.7 (16.9)
**PS format**, *n* (%)			
Liquid	6 (50)	3 (50)	9 (50)
Powder	4 (33)	3 (50)	7 (39)
Combination Liquid & Powder	1 (8.5)	-	1 (5.5)
Combination Liquid & Tablets	1 (8.5)	-	1 (5.5)
**PS characteristics**, *n* (%)			
GMP-PS only	3 (25)	1 (17)	4 (22)
AA-PS only	7 (58)	5 (83)	12 (67)
Combination of GMP-PS + AA-PS	2 (17)	-	2 (11)
Metabolic control **, *n* (%)	**PKU (*n* = 9)**	**TYR (*n* = 6)**	**Total (*n* = 15)**
Within target	7 (78)	-	7 (46)
Above target	2 (22)	2 (33)	4 (27)
Fluctuating around target	-	4 (67)	4 (27)

* Children (13/18, age 3–17 years); adults (5/18, age 18–45 years) ** PKU Target treatment ranges for Phe control: 120–360 µmol/L for children <12 years and <600 µmol/L for children ≥12 years and adults. TYR Target treatment ranges for Phe and Tyr control: Phe > 50 µmol/L and Tyr 200–400 µmol/L for children <12 years. Abbreviations: AA-PS, Amino acid protein substitute; GMP-PS, Glycomacropeptide protein substitute; PKU, phenylketonuria; PS, protein substitute; TYR, tyrosinemia; SD, standard deviation.

## Data Availability

The data presented in this study are available on request from the corresponding author.

## Appendix A

**Table A1:** Nutritional information on the powdered GMP-PS for PKU and TYR patients.

AVERAGE CONTENTS (per 12.5 g Serving)		PKU GMP-PS	TYR GMP-PS
**Energy**	kcal	42	40
	kJ	177	172
**Protein Equivalent**	g	10	10
**Carbohydrate**	g	0.43	0.10
sugars	g	0.04	0.01
**Fat**	g	0	0
**Fibre**	g	0	0
**Minerals**			
sodium	mg (mmol)	141 (6.1)	141 (6.1)
potassium	mg (mmol)	124 (3.2)	124 (3.2)
chloride	mg (mmol)	<8.8 (<0.25)	<8.8 (<0.25)
calcium	mg (mmol)	<16.3 (<0.41)	<16.3 (<0.41)
phosphorus	mg (mmol PO_4_)	40.0 (1.3)	40.0 (1.3)
magnesium	mg (mmol)	0.8 (0.03)	0.8 (0.03)
iron	mg	0.02	0.02
zinc	mg	0.01	0.01
selenium	μg	1.2	1.2
chromium	μg	0.40	0.40
iodine	μg	1.5	1.5
**Amino Acids**			
L-alanine	g	0.40	0.40
L-arginine	g	0.50	0.48
L-aspartic acid	g	0.60	0.59
L-cystine	g	0.29	0.14
L-glutamic acid	g	1.3	1.3
Glycine	g	0.07	0.94
L-histidine	g	0.40	0.49
L-isoleucine	g	0.73	0.72
L-leucine	g	1.5	1.4
L-lysine	g	0.43	0.78
L-methionine	g	0.13	0.23
L-phenylalanine	mg	18.0	18.0
L-proline	g	0.80	0.77
L-serine	g	0.50	0.51
L-threonine	g	1.1	1.1
L-tryptophan	g	0.24	0.24
L-tyrosine	g	1.0	3.5
L-valine	g	0.87	0.88
L-carnitine	mg	10.0	10.0
Taurine	mg	30.0	30.0
**Others**			
osmolality	mOsm/kg H_2_O	170	250
osmolarity	mOsmol/L	160	240
potential renal solute load	mOsmol/L	351	351
